# Stroke in Fabry Disease: Identification of Risk Factors for Stroke in a Large Single‐Centre Cohort

**DOI:** 10.1111/ene.70415

**Published:** 2025-11-07

**Authors:** David Moreno‐Martinez, Sara Lucas‐Del‐Pozo, Lucia Lavalle, Uma Ramaswami, Lionel Ginsberg, Guillem Pintos‐Morell, Derralynn A. Hughes

**Affiliations:** ^1^ Lysosomal Storage Disorders Unit Royal Free Hospital NHS Foundation Trust and University College London London UK; ^2^ Department of Clinical and Movement Neurosciences Queen Square Institute of Neurology and University College London London UK; ^3^ Charles Dent Metabolic Unit The National Hospital for Neurology and Neurosurgery London UK; ^4^ Vall d'Hebron Institute of Research (VHIR) Vall d'Hebron Barcelona Hospital Campus Barcelona Spain

**Keywords:** Fabry disease, lysosomal storage disorders, stroke

## Abstract

**Objectives:**

Risk factors for stroke in Fabry disease (FD) are not precisely known. This study presents a retrospective cohort analysis from one reference centre in the United Kingdom to determine risk factors for stroke and to develop a predictive model.

**Methods:**

Patients > 18 years old were included in the study and were followed from their first visit until March 2019. The main outcome of the survival analysis was time to stroke. The independent risk factors were evaluated using a multivariate Cox regression.

**Results:**

Of 414 patients, 368 were included in the survival analysis. 227 (61.7%) were female, with a median baseline age of 42.7 (IQR 27.8–54.8) for males and 39.9 (26.6–51.2) years for females. 56 (39.7%) males and 64 (28.2%) females had the N215S genotype. 41 patients had a stroke at baseline (11.2%), rising to 69 (18.8%) at the end—66.7% lacunar, 18.9% anterior circulation, 13% posterior circulation and 1.4% venous thrombosis. Median follow‐up was 10.4 years. Median time to stroke in males and females was 43 (43–43) and 58 (58–59) years, respectively. In the multivariate analysis, a concomitant autoimmune disease was associated with an increased risk of stroke, while glomerular filtration rate > 90 and N215S genotype were associated with a decreased risk.

**Interpretation:**

Sex was not associated with stroke, despite males being more severe. These results might help stratify patients and are of interest not only to metabolic physicians, but to general stroke physicians too.

## Graphical abstract

A retrospective cohort study of 368 adults with Fabry disease in the UK analysed time to stroke over approximately 10 years. It found that concomitant autoimmune disease increased stroke risk, while eGFR > 90 mL/min and the p.N215S genotype were associated with lower risk. A nomogram was created for individual risk prediction based on these findings.

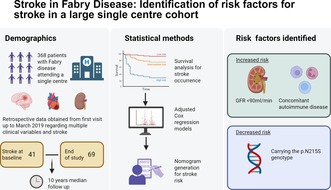

## 1 Introduction

Fabry disease (FD; OMIM#301500) is an X‐linked lysosomal storage disorder caused by pathogenic *GLA* variants, leading to alpha‐galactosidase A deficiency with sphingolipid accumulation, particularly globotriaosylceramide (Gb3) and globotriaosylsphingosine (lyso‐Gb3) [1], with multiorgan dysfunction, especially cardiac and renal. FD affects the central and peripheral nervous systems, causing stroke, white matter lesions, pain and autonomic disturbance [2].

The highly variable clinical presentation has led to the distinction between ‘classical’ and ‘later‐onset’ phenotypes [3], which cannot be predicted exclusively by genotype [4, 5]. Classical FD is multisystemic, with peripheral manifestations such as acroparaesthesia, angiokeratoma and cornea verticillata. Conversely, the later‐onset phenotype is often attenuated and predominantly single‐organ, usually cardiac or renal [3].

Stroke prevalence in FD ranges from approximately 4.3%–48%, varying by stroke's definition, patients' characteristics and sample size [6]. In a cohort study in 2018, 23.8% of patients had a stroke [7]. FD increases stroke risk across all age groups versus controls [8]. A single‐centre study which performed an exome‐based gene panel on 172 patients with juvenile‐onset ischaemic stroke showed that 1.2% carried *GLA* variants [9]. Moreover, FD accounts for roughly 0.5%–10% of strokes of unknown source, depending on inclusion criteria [10].

The pathophysiology of stroke in FD remains unclear. Large‐vessel occlusion (LVO) strokes likely reflect cardiac‐derived embolism, while small vessel disease and lacunar infarcts relate to hypertension and vascular collagenosis [11]. Other potential mechanisms include vessel wall thickening, blood–brain barrier disruption, Gb3‐mediated vascular wall reactivity along with impaired cerebral blood flow [6, 12].

Modifiable factors (e.g., smoking) are common in FD and the general population [13], but shared risks alone cannot explain this higher prevalence. FD‐specific factors may be implicated. Angiotensin pathway polymorphisms and the presence of Factor V Leiden have been associated with stroke risk [14].

Renal and cardiac impairment in FD may increase risk, since decreased glomerular filtration rate (GFR) [15], left ventricular hypertrophy (LVH) and reduced global systolic strain correlate with stroke [7]. However, the evidence is controversial. A study did not demonstrate a relationship between the progression of infarcts of > 3 mm and traditional vascular risk factors or FD‐organ involvement [16], while others linked GFR and CNS damage [17].

This study provides new insights into patients' stratification and suggests mechanisms underlying the pathophysiology of stroke in FD.

## 2 Materials and Methods

### 2.1 Patients and Data Collection

We conducted a retrospective cohort analysis including all patients from a national reference centre for lysosomal storage disorders in the United Kingdom. We used fully anonymised retrospective data from routine clinical care; therefore, informed consent was not required. Adult FD patients were included, defined as older than 18 years with at least one follow‐up visit.

Analysis included all patients eligible in March 2019. Data was extracted for each patient from the first appointment in the reference centre (baseline) to March 2019 (end of study). Data regarding age, genotype, genotype–phenotype correlation, biomarkers, cardiovascular risk factors [hypertension (HTN), diabetes mellitus (DM), smoking, body mass index (BMI)], white matter lesions (WML), stroke, left atrium size (LAS), left ventricular ejection fraction (LVEF), patent foramen ovale (PFO), presence of AF, baseline left ventricular mass index (LVMI), presence of concomitant autoimmune disease, GFR, 24‐h proteinuria, sex, D‐Dimer levels and treatment status were collected.

All genotypes were included. Recent studies suggest that controversial genotypes might be related to neurological involvement in FD [18]. These genotypes, like A143T and R118C, were not excluded, as patients exhibited Fabry‐related symptomatology and their analysis was considered informative regarding potential impact, or lack of it, on stroke. Rather than creating a single variable classifying patients as classical or late‐onset, each clinical manifestation was analysed separately, since criteria for this dichotomy vary in the literature and no standard definition exists.

LysoGb3 was routinely measured in dried blood spot (DBS) samples at an external laboratory (Centogene). Both plasma and leukocyte alpha‐galactosidase (AGAL) activity were measured at baseline; however, due to changes in practice over time, not all patients had both measurements, and some had no LysoGb3 assessment. GFR was measured via chromium EDTA clearance, and 24‐h proteinuria and D‐Dimer levels were collected from routine follow‐up urine and blood tests, respectively. D‐dimer normal range was changed in March 2016 due to a change in the measuring technique. Therefore, D‐dimer was analysed separately before and after March 2016.

Cardiac variables were derived from echocardiography, as cardiac MRI was not available for all patients. LAS was assessed using the Simpson's biplane method, indexed to BSA and classified as normal, mildly dilated, moderately dilated and severely dilated. LVEF was considered reduced if < 50%, following established guidelines [19].

Concomitant autoimmune disease was reported if it appeared as a diagnosis in the electronic medical records by the leading physician or rheumatology specialist.

Although this is a controversial topic in the field [20], patients were classified following the AHA/ASA latest definition of stroke [21] (details in Data S4). Within ischaemic strokes, we further distinguished between lacunar and large‐vessel distribution infarctions, the latter subcategorised by vascular territory (anterior vs. posterior circulation) [22, 23, 24, 25]. This hybrid classification was chosen to facilitate clinically meaningful subgroup analysis. Transient ischaemic attacks (TIA) were not classified as strokes and patients who had a TIA were excluded from the survival analysis (*n* = 12). Only patients with neuroimaging findings were analysed to avoid subjective bias.

1081 MRIs and corresponding neuroradiology reports were reviewed. MRIs were obtained from routine surveillance, not acute stroke assessment. All silent strokes were chronic at detection, and age at MRI was used as the proxy event time in survival analyses. Details about the MRI sequences and data can be found in Data S4.

### 2.2 Statistical Analysis

Statistical analysis was done using STATA 17.1 Continuous variables are reported as mean (with SD) and categorical ones as frequencies. Groups were compared with chi‐square (*χ* ^2^) test or Fisher exact test for categorical variables, and Student's *t*‐test or Wilcoxon rank sum tests for continuous ones. In the survival analysis, the presence of stroke was the failure variable, with multiple failures allowed for patients with more than one event. Patient age was used as the time variable to adjust for the association between stroke and age. Accordingly, survival time was defined as the patient's age at stroke occurrence. Multiple imputation via chained equations method was used to deal with missing data, including in the regression the Nelson–Aalen estimate of the baseline cumulative hazard and the failure variable for stroke following the latest recommendations [26]. LysoGb3 variables and proteinuria could not be imputed due to non‐convergence (there was an average of 213 missing values in LysoGb3 and 24 h proteinuria variables, while it was 13 for the rest). Therefore, they were analysed as raw data on an exploratory basis, without being included in the multivariate analysis. The GFR variable was included as raw data, grouping by quartiles and grouping by higher or lesser than 90 mL/min/1.73 m^2^.

Kaplan–Meier (KM) curves were analysed with the log‐rank test. Variables with significant KM or demographic results, or deemed clinically relevant, were assessed as risk factors using Cox models, with proportional hazards tested via Schoenfeld residuals. Pegunigalsidase alfa was excluded due to low patient numbers.

All Cox models used shared frailty to account for interpatient variability. For each possible risk factor, a univariate Cox regression model and an adjusted model by sex and N215S genotype, given the high frequency of this genetic variant, were performed [27]. Cardiovascular risk factors (including smoking, hypertension and diabetes) were modelled as time‐fixed covariates, defined at baseline, because follow‐up changes were not consistently available in the retrospective dataset. Conversely, Fabry‐specific treatment was modelled as a time‐varying covariate: patients were considered untreated until therapy initiation, after which their person‐time was attributed to the treated category. To account for potential baseline hazard differences between ever‐treated patients and those who remained treatment‐naïve, Cox models were additionally stratified by treatment status. This dual approach incorporated treatment timing while preserving model validity. Variables with significant results (*p* < 0.05) in the adjusted model were included in a multivariate Cox regression. Those remaining significant were considered potential stroke risk factors.

### 2.3 Nomogram Generation

An exploratory nomogram was generated using a code generated for STATA 17.1 [28]. Once the multivariate model was achieved, the Akaike information criterion (AIC) was compared by adding or subtracting clinically important variables due to their relation to stroke (WML Fazekas score, GFR quartiles, presence of acroparaesthesia, hypertension and AF) using raw data. The model with the lower AIC was selected.

## 3 Results

### 3.1 Description of the Cohort

From a population of 414 FD patients, 34 were excluded for missing data or lack of follow‐up, and 12 for prior TIA (2 of whom also had missing data, included in the 34). Demographic data for all 414 patients are provided in Data S1 and differed little from those of the 368 FD patients final cohort. Table 1 displays demographic data by sex and stroke status for the cohort of 368 patients. Data S3 shows all genetic variants of patients who had a stroke. The analysis presented in the manuscript was conducted in the 368 patients cohort. Only Data S1 refers to the 414 patients cohort.

**TABLE 1:** Demographic data of the survival analysis cohort (*N*: 368).

Variable	Male	Female	*p*	No stroke	Stroke	*p*
Demographics and biochemical						
*N* (%)	141 (38.3)	227 (61.7)				
Baseline stroke, *N* (%)				327 (88.8)	41 (11.2)	
End of study stroke, *N* (%)				299 (81.3)	69 (18.8)	
Age at baseline [years, median (IQR)]	42.7 (27.8–54.8)	39.9 (26.6–51.2)	0.2	36.9 (25.3–50.8)	47.2 (40.1–61.1)	0.001
Age at stroke [years, median (IQR)]	50.6 (41.8–60.8)	51.4 (39.3–68.1)	0.3			
Age at FD diagnosis [years, median (IQR)]	35.1 (16.2–56.2)	34.0 (24.2–45.2)	0.68	31.5 (19–45)	44.1 (28–60.2)	0.005
N215S variant			0.02			0.001
Yes	56 (39.7)	64 (28.2)		109 (36.5)	11 (16.0)	
No	85 (60.3)	163 (71.8)		190 (63.5)	58 (84.0)	
Plasma AGAL (nmol/h/mg, mean [SD])	0.35 (0.7)	4.02 (1.9)	0.001	2.7 (2.4)	2.4 (2.2)	0.3
Leukocyte AGAL (nmol/h/mg, mean [SD])	4 (5.6)	38.2 (20.9)	0.001	25.2 (24.2)	25.0 (22.3)	0.9
LysoGb3 baseline (nmol/L, mean [SD])	36.3 (35.3)	5.3 (4.0)	0.001	12.8 (23.4)	32.1 (32.0)	0.001
Urinary LysoGb3 baseline (pmol/mmol, mean [SD])	8.0 (9.9)	1.5 (1.2)	0.001	3.3 (7.0)	6.8 (6.6)	0.03
Vascular risk factors						
Hypertension, *N* (%)	25 (17.7)	32 (14.1)	0.3	39 (13.0)	18 (31.6)	0.007
Diabetes, *N* (%)	4 (2.8)	9 (4.0)	0.6	9 (3.0)	4 (5.8)	0.3
Smoking, *N* (%)	8 (5.7)	14 (6.0)	0.9	18 (6.0)	4 (5.8)	0.9
D‐Dimer						
Before 2016	109.3 (134.4)	119.9 (118.0)	0.04	107.1 (110.2)	149.1 (163.1)	0.08
After 2016	310.5 (282.9)	196.5 (155.4)	0.05	228.9 (189.5)	371.0 (408.9)	0.7
Renal						
GFR baseline (mL/min/1.73 m^2^, mean [SD])	86.6 (26.7)	93.7 (22.7)	0.008	94.5 (23.2)	76.2 (25.0)	0.001
GFR end (mL/min/1.73 m^2^, mean [SD])	80.3 (32.5)	89.2 (24.0)	0.003	89.8 (27.0)	68.8 (25.0)	0.001
Proteinuria baseline (g/24 h, mean [SD])	0.40 (0.1)	0.20 (0.02)	0.003	0.28 (0.5)	0.29 (0.4)	0.9
Proteinuria end (g/24 h, mean [SD])	0.50 (0.9)	0.19 (0.3)	0.001	0.30 (0.4)	0.30 (0.7)	0.9
Cardiac						
AF			0.001			0.001
No	103 (73.1)	203 (89.4)		259 (86.6)	47 (68.1)	
Yes	36 (25.5)	23 (10.2)		38 (12.6)	21 (30.4)	
PFO			0.9			0.001
Yes	3 (2.1)	5 (2.2)		1 (0.3)	7 (10.1)	
No	138 (97.9)	222 (97.8)		298 (99.7)	62 (89.9)	
LA at end			0.001			0.1
Normal	55 (39.1)	149 (65.6)		174 (58.1)	30 (43.5)	
Mild	44 (31.2)	39 (17.2)		63 (21.1)	20 (29.0)	
Moderate	20 (14.2)	12 (5.3)		22 (7.4)	10 (14.5)	
Severe	8 (5.6)	7 (3.1)		12 (4.0)	3 (4.3)	
LVMI baseline (g/m^2^, mean [SD])	51.5 (24.6)	35.7 (20.6)	0.001	38.9 (22.6)	52.2 (24.2)	0.002
Neurological						
Stroke type at end			0.2			
No stroke	109 (77.2)	190 (83.8)				
Lacunar	19 (13.5)	27 (11.9)				
Anterior	6 (4.3)	7 (3.0)				
Posterior	6 (4.3)	3 (1.3)				
Venous thrombosis	1 (0.7)	0				
WML baseline			0.5			0.001
No WML	81 (57.5)	145 (63.9)		208 (69.5)	18 (26.1)	
Fazekas 1	45 (31.9)	60 (26.4)		72 (24.1)	33 (47.8)	
Fazekas 2	15 (10.6)	22 (9.7)		19 (6.4)	18 (26.1)	
WML end			0.7			0.001
No WML	74 (52.5)	129 (56.8)		192 (64.2)	11 (15.9)	
Fazekas 1	46 (32.6)	65 (28.6)		79 (26.4)	32 (46.3)	
Fazekas 2	17 (12.0)	29 (12.8)		25 (8.4)	21 (30.4)	
Fazekas 3	4 (2.9)	4 (1.8)		3 (1.0)	5 (7.4)	
Acroparaesthesia			0.08			0.003
Yes	63 (43.8)	78 (34.8)		106 (35.5)	38 (44.9)	
No	81 (56.2)	146 (65.2)		193 (64.5)	31 (55.1)	
Other						
Angiokeratoma			0.001			0.001
Yes	51 (36.2)	43 (18.9)		64 (21.4)	30 (43.5)	
No	90 (63.8)	184 (81.1)		235 (78.6)	39 (56.5)	
Concomitant autoimmune disease			0.4			0.01
Yes	5 (3.6)	12 (5.3)		10 (3.3)	7 (10.1)	
No	136 (96.4)	215 (94.7)		289 (96.7)	62 (89.9)	
Treatments						
Initial treatment			0.001			0.002
No treatment	11 (7.8)	106 (46.7)		109 (36.5)	8 (11.6)	
Agalsidase α	90 (63.8)	98 (43.2)		143 (47.8)	45 (65.2)	
Agalsidase β	24 (17.0)	17 (7.5)		29 (9.7)	12 (17.4)	
Migalastat	15 (10.7)	6 (2.6)		17 (5.7)	4 (5.8)	
Pegunigalsidase α	1 (0.7)	0		1 (0.3)	0	
Statins at end			0.001			0.001
Yes	48 (34.0)	40 (17.6)		55 (18.4)	33 (47.8)	
No	93 (66.0)	187 (82.4)		244 (81.6)	36 (52.2)	
Beta‐blocker at end			0.001			0.09
Yes	106 (75.2)	25 (11.0)		44 (14.7)	16 (23.2)	
No	35 (24.8)	202 (89.0)		255 (85.3)	53 (76.8)	
ACEi/ARAII at end			0.008			0.001
Yes	53 (37.6)	56 (24.7)		77 (25.7)	32 (46.4)	
No	88 (62.4)	171 (75.3)		222 (74.3)	37 (53.6)	
Anticoagulants at end			0.001			0.001
None	109 (77.3)	206 (90.8)		268 (89.7)	47 (68.1)	
Warfarin	14 (9.9)	13 (5.7)		14 (4.7)	13 (18.9)	
Apixaban	6 (4.3)	1 (0.4)		6 (2.0)	1 (1.5)	
Dabigatran	3 (2.1)	0		2 (0.7)	1 (1.5)	
Rivaroxaban	9 (6.4)	7 (3.1)		9 (3.0)	7 (10.0)	
Antiplatelets at end			0.008			0.001
None	94 (66.7)	176 (77.5)		237 (79.3)	33 (47.8)	
AAS	35 (24.8)	27 (11.9)		47 (15.7)	15 (21.7)	
Clopidogrel	7 (5.0)	18 (7.9)		9 (3.0)	16 (23.2)	
AAS + Clopidogrel	1 (0.7)	4 (1.8)		1 (0.3)	4 (5.8)	
Missing	4 (2.8)	2 (0.9)		5 (1.7)	1 (1.5)	

Overall, almost two thirds of patients were women (62%). Median baseline age was 42.7 years (IQR 27.8–54.8) for men and 39.9 (IQR 26.6–51.2) for women; age at diagnosis: 35.1 (16.2–56.2) vs. 34.0 (24.2–45.1); age at first stroke: 50.6 (41.8–60.8) versus 51.4 (39.3–68.1). However, baseline age was higher in patients who had a stroke versus those who did not, 36.9 (25.3–50.8) to 44 (28.6–60.2) years. The median follow‐up time was approximately 10 years.

Baseline plasma and leukocyte AGAL differed by sex: plasma AGAL 0.35 ± 0.7 vs. 4.02 ± 1.9 nmol/mg/h, leukocyte AGAL 4.0 ± 5.6 vs. 38.2 ± 20.9 nmol/mg/h (males vs. females). However, no differences were observed between stroke and non‐stroke patients in plasma AGAL (2.7 ± 2.4 vs. 2.4 ± 2.2) or leukocyte AGAL (25.2 ± 24.2 vs. 25.0 ± 22.3), and the results remained non‐significant after stratification by sex. Most patients carried a missense variant (80.1% of males and 78.9% of females), with two‐fifths of males (39.7%) and slightly more than a quarter of females (28.2%) presenting the N215S variant. Despite being so widespread, N215S was found in only 11% of stroke patients, compared with 36.5% of those without.

Clinical variables more frequent or severe in males included angiokeratoma, GFR at baseline and the end, baseline LVMI, baseline and final LAS, AF at the end and baseline, and final proteinuria. Interestingly, acroparesthesia, WML and stroke were not more prevalent in males, despite their typical association with classical FD.

At baseline, treatment status by sex (male versus female) was: no treatment (7.8% vs. 46.7%), Agalsidase alpha (63.8% vs. 43.2%), Agalsidase beta (17% vs. 7.5%), Migalastat (10.7% vs. 2.6%) and one on Pegunigalsidase alpha (0.7%–0%).

### 3.2 Demographics of Stroke

Among the 368 patients in the main analysis, 41 presented a stroke at baseline, increasing to 69 patients (18.8%) by the study end. Stroke distribution by sex was lacunar (13.5% in men vs. 11.9% in women), anterior circulation (4.3% vs. 3.0%), posterior circulation (4.3% vs. 1.3%) and venous thrombosis (0.7% vs. 0%), with no overall sex predominance. Additionally, 42% of strokes were clinically silent, detected only on the MRI.

Variables significantly more prevalent or pronounced in stroke patients included older baseline and FD‐diagnosis age, non‐N215S genotype, higher baseline plasma and urinary lyso‐Gb3, angiokeratoma, acroparesthesia, concomitant hypertension, lower baseline and final GFR, AF, higher baseline LVMI, baseline and final WML, autoimmune disease and PFO. Use of statins, beta‐blockers, ACEi/ARB, antiplatelets and anticoagulants was also associated with stroke, although this association likely reflects secondary prevention following the event. Results are displayed in Table 1.

### 3.3 Stroke‐Free Survival Analysis and Univariate Analysis

The survival analysis time spans from 15 years old to 87. No stroke was observed before the age of 26. The estimated median survival times for men and women were 43 (0.1) and 58 (0.1) years old.

**FIGURE 1:**
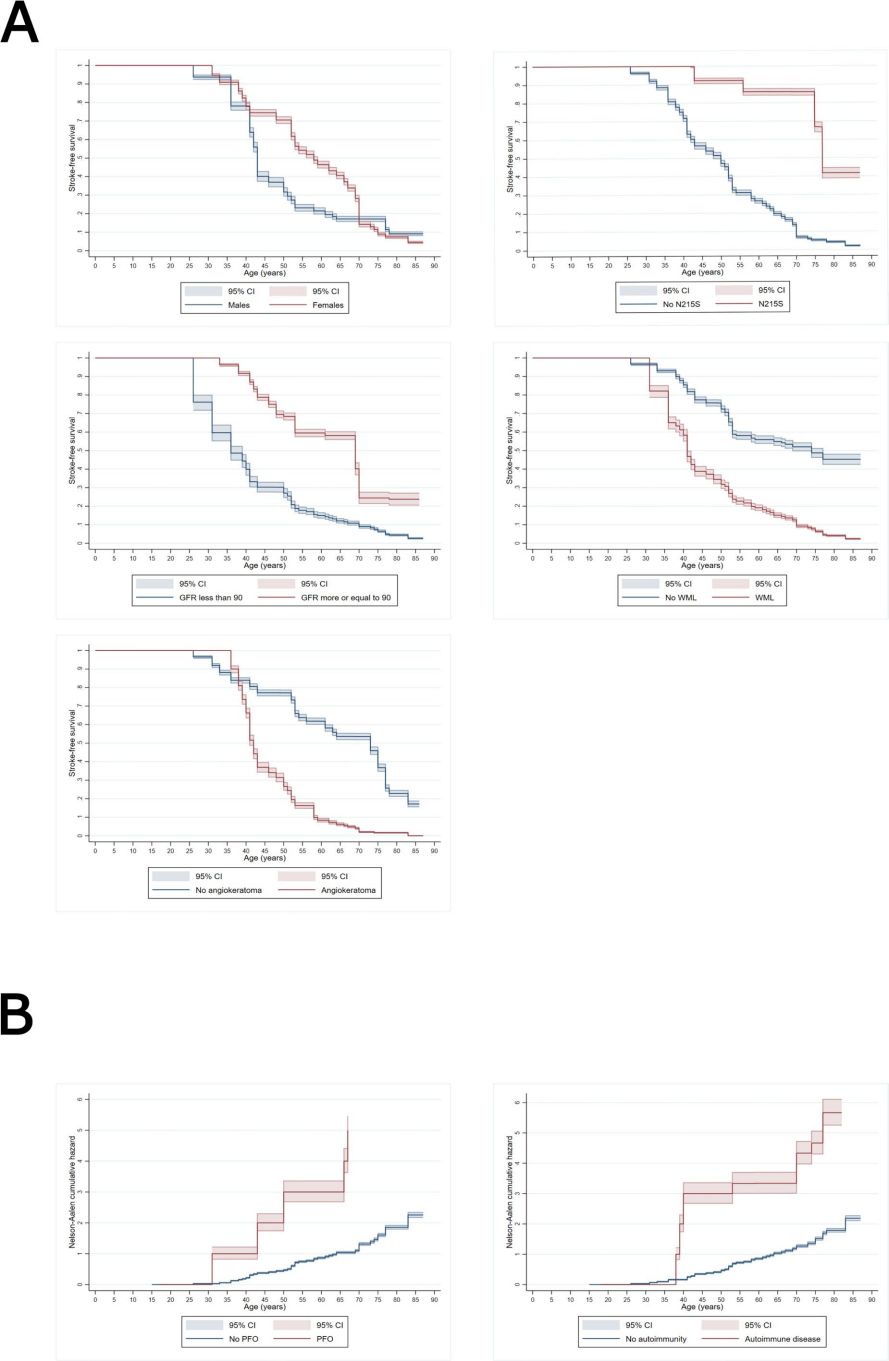
Survival and Hazard curves. (A) Kaplan–Meier curves. (B) Nelson–Aalen Hazard estimates for the variables PFO and autoimmunity as given the low number of patients with those conditions it allows a better visualisation of the risk.

Potential risk/protective factors for stroke in the univariate analysis included (hazard ratio, 95% CI): N215S genotype (0.33, 0.17–0.65), nonsense versus missense genetic variant (2.3, 1.23–4.19)—but when removing N215S from the missense list, nonsense variants lost their significance, PFO (7.5, 3.8–15), overall presence of WML (2.7, 1.2–6), Fazekas 1 versus no WML (2.6, 1–5.9), Fazekas 2 versus no WML (3.1, 1.1–8.8), higher GFR (0.98, 0.97–0.99), GFR > 90 (0.33, 0.16–0.68), GFR between 0 and 74 versus > 107 (4.4, 1.2–16.4), autoimmune disease (3.4,1.4–8), acroparaesthesia (2, 1.1–3.5), angiokeratoma (2.4, 1.4–4.2), enzyme replacement therapy (ERT) versus Migalastat (3.1, 1.4–7.2), Migalastat versus agalsidase‐alpha (0.3, 0.13–0.8) (Table 2 and Figure 1). Antiplatelet and anticoagulant use were excluded as risk factors, since they represent expected secondary prevention after stroke. Naïve status HR could not be estimated due to the violation of the proportional hazard assumption.

**TABLE 2:** Hazard ratios from the different models (cohort size n: 368).

Variable	Unadjusted HR	CI	*p*	Adjusted HR	CI	*p*
Demographics and genetics						
Sex (female)	0.95	0.53–1.72	0.9	0.7	0.36–1.33	0.28
N215S	0.33	0.17–0.65	0.001	0.3	0.14–0.6	0.001
Mutation type						
Missense versus nonsense	2.3	1.23–4.19	0.009	1.6	0.89–3	0.13
Missense versus rearrangement	1.5	0.6–4	0.4	1.1	0.43–3.1	0.8
Mutation type without N215S						
Nonsense versus missense	1.7	0.9–3.1	0.1	1.6	0.86–3.1	0.1
Rearrangement versus missense	1.1	0.42–2.9	0.2	1.1	0.4–3	0.9
LysoGb3 Δ	1.2	1.1–1.3	0.001	1.1	1–1.2	0.003
Urinary LysoGb3 Δ	1.8	1.2–2.5	0.001	2.11	0.8–5	0.1
Cardiac						
Dilated LA (yes/no)^a^	1	0.5–2	0.9	0.86	0.4–1.8	0.7
LVH yes/no	1.1	0.6–2.2	0.7	1	0.55–2.1	0.2
LVMI value	1	0.98–1	0.9	1	0.99–1	0.9
LA status						
Mildly versus non dilated	1.1	0.5–2.3	0.7	0.96	0.45–2	0.9
Moderately versus non dilated	0.82	0.2–2.8	0.7	0.65	0.19–2.3	0.5
Severely versus non dilated	—	—	—	—	—	—
Atrial fibrillation	1.2	0.6–2.5	0.6	0.9	0.4–2	0.8
PFO	7.5	3.8–15	< 0.001	7.3	3.4–15.6	< 0.001
Neurological						
WML yes/no	2.7	1.2–6	0.02	2.5	1.1–5.5	0.03
WML status						
Fazekas 1 versus no WML	2.6	1–5.9	0.03	2.4	1.1–5.5	0.03
Fazekas 2 versus no WML	3.1	1.1–8.8	0.03	2.6	0.93–7	0.07
Acroparaesthesia	2	1.1–3.5	0.02	1.5	0.8–2.7	0.2
Renal						
GFR	0.98	0.97–0.99	0.005	0.98	0.98–1	0.08
GFR > 90	0.33	0.16–0.68	0.003	0.4	0.19–0.81	0.012
GFR quartile						
> 75% versus 0%–25% (> 107 vs. 92–106)	1.6	0.4–6	0.7	1.3	0.35–5	0.7
> 75% versus 25%–50% (> 107 vs. 74–91)	3.4	0.9–12.9	0.07	2.76	0.7–10.4	0.1
> 75% versus 50%–75% (> 107 vs. 0–74)	4.4	1.2–16.4	0.02	3.3	0.85–13	0.08
Proteinuria Δ	0.9	0.9–1.01	0.4	0.9	0.9–1.1	0.4
Cardiovascular risk factors						
Hypertension	0.97	0.4–2	0.9	0.9	0.4–2	0.8
Diabetes	0.6	0.2–1.6	0.3	0.6	0.2–1.8	0.3
Smoking	0.9	0.3–2.3	0.7	1.1	0.4–2.7	0.9
Statins	1	0.5–2.2	0.9	1.1	0.6–2.3	0.7
ACEI or ARAII	0.8	0.4–1.4	0.5	0.7	0.4–1.2	0.2
Beta blocker	0.75	0.4–1.4	0.4	0.8	0.4–1.5	0.5
Treatment						
Treatment naïve^a^			0.01			
Beta versus alpha	1.7	0.8–3.6	0.2	1.6	0.7–3.2	0.2
Migalastat versus alpha	0.3	0.13–0.8	0.02	0.4	0.15–1	0.05
Alpha versus beta	0.6	0.3–1.2	0.2	0.6	0.3–1.3	0.2
Migalastat versus beta	0.3	0.11–0.7	0.06	0.6	0.2–1.5	0.3
ERT versus migalastat	3.1	1.4–7.2	0.008	2.4	0.98–5.6	0.05
Other						
Angiokeratoma	2.4	1.4–4.2	0.001	2	1.2–3.5	0.01
Autoimmune disease	3.4	1.4–8	0.006	3.5	1.4–8.7	0.007
D‐dimer pre2016	1	0.99–1	0.1	1	0.99–1	0.08
D‐dimer after 2016	1	0.99–1	0.7	1	0.99–1	0.6

^a^ Due to the violation of the proportional hazards assumption, the significance of the log‐rank test is presented Δ the HR calculus was done in the non‐imputed cohort.

### 3.4 Adjusted Model and Multivariate Analysis

When adjusted by sex and N215S genotype (Table 2), variables associated with increased or reduced risk of stroke were (HR, 95% CI): N215S genetic variant (0.3, 0.14–0.6), PFO (7.3, 3.4–15.6), presence of WML (2.5, 1.1–5.5), Fazekas 1 versus no WML (2.4, 1.1–5.5), GFR > 90 (0.4, 0.19–0.81), autoimmune disease (3.5, 1.4–8.7) and angiokeratoma (2, 1.2–3.5). Interestingly, both plasma and urinary lysoGb3 were associated with stroke even after adjustment but could not be included in the multivariate model as noted above.

Results of the multivariate analysis are shown in Table 3. To avoid repetition, overall WML was included, without a Fazekas grade. Given the low number of patients with PFO and the absence of systematic screening, it was omitted from the multivariate model. In this model, variables independently associated with stroke risk were: N215S (0.4, 0.2–0.8), autoimmunity (3.3, 1.4–7.4) and GFR higher than 90 (0.5, 0.2–0.9). No association was found for sex, WML, or angiokeratoma, although the latter two showed a non‐significant trend towards association with stroke. The *χ* ^2^ of the relationship between the presence of WML and GFR higher or lower than 90 was 53.9 (*p* < 0.0001) and, therefore, the analysis was done by subtracting either GFR or WML variables. When repeating the model without GFR, WML *p* value was 0.05. However, the CI included 1. Consequently, it was deemed only as a trend. Similarly, in the analysis without WML, angiokeratoma *p*‐value was 0.04, including 1 in the CI, precluding its definition as a stroke risk factor. All other variables remained unchanged.

**TABLE 3:** Multivariate analysis results (cohort size n:368).

Model	Variable	Hazard ratio	CI	*p*
Complete				
	N215S	0.4	0.2–0.8	0.007
	Gender (female)	0.8	0.4–1.4	0.4
	Autoimmunity	3.3	1.4–7.4	0.005
	Angiokeratoma	1.6	0.9–3	0.09
	GFR > 90	0.5	0.2–0.9	0.03
	WML presence	2	0.9–4.5	0.1
Without GFR				
	N215S	0.36	0.2–0.7	0.004
	Gender (female)	0.67	0.4–1.2	0.2
	Autoimmunity	3.1	1.3–7.3	0.008
	Angiokeratoma	1.7	1–3.1	0.05
	WML presence	2.2	1–5	0.05
Without WML				
	N215S	0.4	0.2–0.8	0.006
	Gender (female)	0.8	0.4–1.4	0.4
	Autoimmunity	3.4	1.5–7.6	0.003
	Angiokeratoma	1.8	1–3.1	0.04
	GFR > 90	0.4	0.2–0.8	0.01

### 3.5 Predictive Nomogram

The AIC of the complete multivariate model of Table 3 was 142. Ultimately, the model with the lowest AIC included acroparaesthesia, PFO, as well as GFR and WML as dichotomous variables. The AIC of that model was 71. However, PFO was excluded from the nomogram because not all patients were actively screened, which could introduce ascertainment bias.

**FIGURE 2:**
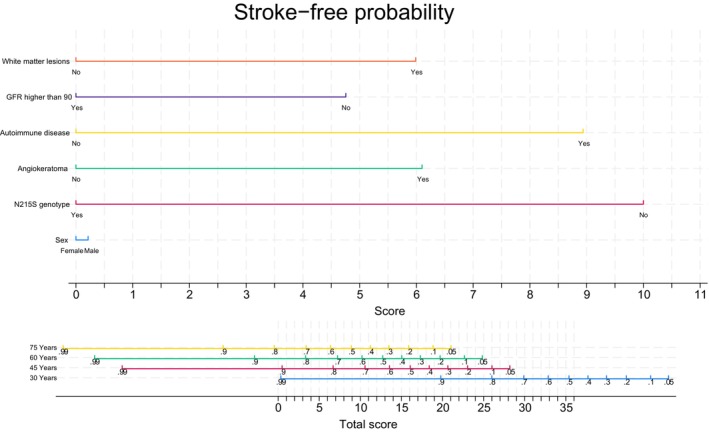
Nomogram. The nomogram was created from the best AIC multivariate model to obtain stroke‐free survival probability at a given age.

The nomogram (Figure 2) visually represents the model and estimates stroke‐free survival by age; detailed instructions are in Data S2.

## 4 Discussion

This observational study of 368 FD patients is one of the largest studies exploring stroke risk factors in FD, an area where controversial results have been published. Additionally, this study is one of the few where all the variables were controlled by age. Our results show that, in this cohort, stroke happened in approximately a fifth of patients, almost doubling the baseline prevalence. This finding agrees with published longitudinal studies [16], and is relevant due to the relationship between CNS infarction and cognitive decline [29].

Strokes did not display a sex predominance in distribution and median survival time, raising the hypothesis that the nervous system might be affected in all patients independently of the X chromosome skewing. Interestingly, acroparaesthesia had no sex predominance either. While some research has already demonstrated the independence of stroke from sex [30], other studies found that males have an increased risk of stroke [16]. This highlights that different FD patients' cohorts may have a differential risk for stroke depending on epidemiological and genetic factors.

As stated above, this study described strokes using a hybrid system combining infarct pattern and vascular territory, reflecting the physiopathology of Fabry disease and allowing comparability with prior studies. Two‐fifths of strokes were clinically silent, which may influence recommended neuroimaging follow‐up protocols. Based on this and previous studies [16], guidelines on neuroimaging intervals in FD should undergo expert review.

Additionally, most strokes were lacunar, suggesting embolic sources may not play a major role in FD stroke physiopathology. Recent evidence shows stroke recurrence in embolic stroke of undetermined source is associated with elevated D‐dimer levels [31]. In our study, however, D‐dimer was not associated with stroke occurrence, pointing towards an alternative physiopathology distinct from embolic mechanisms. Similarly, no association was found between AF and stroke in this study, suggesting limited involvement of embolic mechanisms in stroke in FD.

However, the significant association between stroke and PFO evidenced in this study appears to contradict the previous finding. Without revisiting the ongoing debate regarding the relevance of PFO in stroke [32], it is notable that in our study two of seven stroke patients with PFO (28.57%) had a lacunar infarct. Nonetheless, since patients without stroke were not routinely assessed for PFO, this may have introduced bias, potentially inflating PFO's apparent association with stroke and amplifying its perceived significance. Hence, PFO was excluded from the final model.

Beyond conventional cardiovascular risk factors for stroke already studied [13], a triad of endothelial dysfunction, increased reactive oxygen species (ROS) and immunological abnormalities with an increased thrombotic state has been reported as a potential causative agent of CNS pathology in FD [33, 34]. To that point, we tried to include variables that indirectly represented aspects of the triad, like concomitant autoimmune disease and D‐dimer levels, as well as other manifestations that might be pathophysiologically linked with stroke, like LAS, acroparaesthesia, or WML. The associations of concomitant autoimmune disease (see Data S1 for specific conditions), GFR impairment and WML with increased stroke risk in FD support this triad concept through their contribution to endothelial dysfunction. While autoimmunity has increasing published evidence each year [35], elucidating a causative role or association of GFR and WML with stroke in FD remains more challenging.

GFR and WML have been associated in the general population [36]. This connection influences stroke risk through a mechanism yet unknown [37]. However, our analysis cannot exclude a direct effect of WML in the occurrence of stroke since FD's WML might stem from different mechanisms than in the general population, potentially enhancing stroke risk. This warrants further investigation into the singular contribution of FD‐related WML to stroke pathophysiology.

From a cardiac perspective, no variable other than PFO was associated with stroke, although previous studies have suggested links between left ventricular hypertrophy and global systolic dysfunction [7]. Furthermore, the late‐onset cardiac variant N215S, despite its high cardiac burden, appeared protective against stroke in our study. Although previously reported [38], that study only analysed clinical events, therefore missing the correlation of neuroimaging and silent infarcts, which we show to be frequent. Even with silent infarcts included, this genotype remained associated with a lower stroke risk in our analysis. This may reflect the overall lesser severity of patients with this genetic variant; however, since their phenotype is predominantly cardiac and this effect is absent in other missense genotypes, it raises the possibility of a largely extra‐cardiac aetiology of stroke in FD.

Although this cohort had a high prevalence of N215S patients, the statistical analysis adjusted and accounted for this factor, ensuring it was controlled [27]. A note of caution is warranted. The statistical effect in our model shows that N215S patients develop fewer strokes but are not fully protected. Therefore, we conclude that their monitoring strategy must be the same as the other FD patients, as they still are at risk of neurovascular events.

Strokes occurred in carriers of variants with disputed pathogenicity (p.A143T, p.R118C; Data S3), underscoring the need for ongoing clinical follow‐up of VUS.

Migalastat was associated with fewer strokes than agalsidase alfa (and almost significant versus beta) in the univariate analysis, increasing its *p*‐value to 0.05 after adjustment, suggesting confounding factors. Notably, 36% of N215S patients, a variant associated with fewer strokes in the study, were on migalastat at study end, versus 16.2% of non‐N215S. Nevertheless, this spur effect might encourage further studies on CNS‐penetrant small molecules and appropriately designed studies to evaluate CNS treatment effects.

Our findings suggest that stroke in FD arises from complex, multifactorial mechanisms. Although not yet validated for clinical use, the nomogram may support clinical reasoning and patient stratification. Prospective studies are needed to validate its utility and inform preventive and therapeutic strategies.

## 5 Limitations

This study has several limitations. Its retrospective design precludes causal inference, and the predominance of the N215S variant may limit generalisability. Although large for a rare lysosomal disorder, the sample size remains smaller than in studies typically assessing risk factors.

All silent strokes were detected incidentally on routine surveillance MRI and were chronic at discovery. Because lesion age was indeterminate, we used age at MRI as a proxy in survival analyses, which likely assigns events to a later time, potentially underestimating statistical effects. This is a common limitation in silent infarct studies. Advanced imaging could improve lesion dating in future work.

## 6 Conclusion

Stroke pathophysiology in FD remains unclear. Adjusted analysis showed lower GFR and a concomitant autoimmune disease increased stroke risk, while carrying a N215S genotype reduced it. WML and angiokeratoma presence showed a trend towards increased risk. Findings suggest multiple pathways may contribute to stroke in FD, warranting further study.

## Author Contributions

Conceptualisation: David Moreno‐Martinez, Sara Lucas‐Del‐Pozo, Lionel Ginsberg, Guillem Pintos‐Morell and Derralynn A. Hughes. Methodology: David Moreno‐Martinez, Sara Lucas‐Del‐Pozo, Uma Ramaswami, Lionel Ginsberg, Guillem Pintos‐Morell and Derralynn A. Hughes. Software, data curation and investigation: David Moreno‐Martinez, Sara Lucas‐Del‐Pozo and Derralynn A. Hughes. Validation: David Moreno‐Martinez, Sara Lucas‐Del‐Pozo, Guillem Pintos‐Morell, Lucia Lavalle and Derralynn A. Hughes. Formal Analysis: David Moreno‐Martinez, Sara Lucas‐Del‐Pozo and Derralynn A. Hughes. Supervision: Derralynn A. Hughes. Funding Acquisition: David Moreno‐Martinez, Sara Lucas‐Del‐Pozo and Derralynn A. Hughes. Visualisation: David Moreno‐Martinez, Sara Lucas‐Del‐Pozo, Lucia Lavalle, Uma Ramaswami, Lionel Ginsberg, Guillem Pintos‐Morell and Derralynn A. Hughes. Project Administration: David Moreno‐Martinez and Derralynn A. Hughes. Resources: David Moreno‐Martinez, Sara Lucas‐Del‐Pozo, Lucia Lavalle and Derralynn A. Hughes. Writing: David Moreno‐Martinez, Sara Lucas‐Del‐Pozo, Derralynn A. Hughes, Lucia Lavalle, Uma Ramaswami, Lionel Ginsberg and Guillem Pintos‐Morell.

## Conflicts of Interest

D.A.H. received honoraria for speaking and advisory boards from Takeda, Sanofi, Amicus, Freeline, Chiesi, Uniqure, Acelink and Idorsia. D.M.‐M. received honoraria for speaking and travel grants from Sanofi, Takeda and Amicus. S.L.‐D.‐P. received honoraria for speaking and travel grants from Sanofi, Takeda and Amicus. G.P.‐M. received honoraria for speaking, advisory boards and travel grants from Takeda and Amicus. U.R. received honoraria for lectures and/or advisory boards from Amicus, Chiesi, Sanofi and Takeda; research grants from Amicus, Chiesi, Intrabio and Takeda. L.G. received honoraria and travel grants for speaking and advisory boards from Takeda. L.L. declare no conflicts of interest.

## Supporting information


**Data S1:** Characteristics of the full 414 cohort.

**Data S2:** How to use and interpret the nomogram.

**Data S3:** Genetic variants of stroke patients.

**Data S4:** Stroke classification criteria.

## Data Availability

The data that support the findings of this study are available on request from the corresponding author. The data are not publicly available due to containing information that could compromise patient privacy.
