# Supplementary material for: Stroke in Fabry Disease: Identification of Risk Factors for Stroke in a Large Single‐Centre Cohort
Source: Eur J Neurol. 2025 Nov 7;32(11):e70415. doi: 10.1111/ene.70415 (PMC12593542; doi:10.1111/ene.70415)
Supplement: Supplementary file 1 — Data S1: Characteristics of the full 414 cohort. [file ENE-32-e70415-s002.docx]

| Section / Variable | Male | Female | p-value |
| --- | --- | --- | --- |
| **Demographics & Biochemical** |  |  |  |
| N (%) | 157 (38) | 257 (62) |  |
| Age of diagnosis [years, mean (SD)] | 36.4 (21.2) | 35.9 (16.3) | 0.97 |
| Baseline age [years, mean (SD)] | 42.3 (16.4) | 40.3 (16.5) | 0.17 |
| **Baseline age groups [n, column %]** |  |  |  |
| 10 to 20 years old | 20 (12.7) | 37 (14.4) |  |
| 21 to 30 years old | 26 (16.6) | 49 (19.1) |  |
| 31 to 40 years old | 25 (16) | 47 (18.3) |  |
| 41 to 50 years old | 35 (22.3) | 58 (22.5) |  |
| 51 to 60 years old | 28 (17.8) | 37 (14.4) |  |
| 61 to 70 years old | 17 (10.8) | 17 (6.6) |  |
| 71 to 80 years old | 6 (3.8) | 11 (4.3) |  |
| 81 to 90 years old | 0 (0) | 1 (0.4) |  |
| Total | 157 (100) | 257 (100) | 0.7 |
| Age at the end [years, mean (SD)] | 52.9 (16.7) | 50.4 (16.9) | 0.08 |
| Time in the study [years, mean (SD)] | 10.6 (5.69) | 10.2 (4.7) | 0.7 |
| Plasma AGAL [nmol/h/mg, mean (SD)] | 0.34 (0.7) | 4.05 (1.9) | <0.0001 |
| Leukocyte AGAL [mmol/h/mg, mean (SD)] | 4.29 (5.6) | 37.8 (20.8) | <0.0001 |
| **Type of mutation [N (column %)]** |  |  |  |
| Missense | 117 (74.5) | 186 (72.4) |  |
| Nonsense | 16 (10.2) | 28 (10.9) |  |
| Rearrangement | 13 (8.3) | 21 (8.2) |  |
| Missing | 11 (7) | 22 (8.6) |  |
| Total | 157 (100) | 257 (100) | 0.9 |
| **N215S genetic variant [N (column %)]** |  |  |  |
| Yes | 58 (36.9) | 64 (24.9) |  |
| No | 88 (56.1) | 171 (66.5) |  |
| Missing | 11 (7) | 22 (8.6) |  |
| Total | 157 (100) | 257 (100) | 0.03 |
| Baseline LysoGb3 [nmol/L, mean (SD)] | 35.9 (34.4) | 5.24 (4.03) | <0.0001 |
| Baseline Urinary LysoGb3 [pmol/mmol creatinine, mean (SD)] | 7.81 (9.64) | 1.51 (1.24) | <0.0001 |
| BMI [kg/m², mean (SD)] | 25.7 (5.56) | 25.4 (4.6) | 0.92 |
| **Vascular Risk Factors** |  |  |  |
| **Diabetes [N, column %]** |  |  |  |
| Yes | 5 (3.18) | 6 (2.33) |  |
| No | 81 (51.59) | 137 (53.31) |  |
| Missing | 71 (45.22) | 114 (44.36) |  |
| Total | 157 (100) | 257 (100) | 0.8 |
| **Hypertension [N, column %]** |  |  |  |
| Yes | 19 (12.1) | 24 (9.34) |  |
| No | 67 (42.68) | 120 (46.69) |  |
| Missing | 71 (45.22) | 113 (43.97) |  |
| Total | 157 (100) | 257 (100) | 0.57 |
| **Smoking [N, column %]** |  |  |  |
| Yes | 8 (5.1) | 16 (6.23) |  |
| No | 78 (49.68) | 128 (49.81) |  |
| Missing | 71 (45.22) | 113 (43.97) |  |
| Total | 157 (100) | 257 (100) | 0.88 |
| **D-Dimer [ng/mL, mean (SD)]** |  |  |  |
| Before March 2016 | 110.6 (127.7) | 118.1 (111.9) | 0.07 |
| After March 2016 | 343.8 (280.7) | 268.7 (151.1) | 0.63 |
| **Renal Parameters** |  |  |  |
| Baseline GFR [ml/min/m², mean (SD)] | 85.1 (26.8) | 93.1 (23) | 0.003 |
| GFR at the end [ml/min/m², mean (SD)] | 78.9 (32.2) | 88.7 (24.2) | 0.0012 |
| Baseline proteinuria [mg/24h, mean (SD)] | 0.41 (0.62) | 0.2 (0.31) | 0.001 |
| Proteinuria at the end [mg/24h, mean (SD)] | 0.51 (0.9) | 0.18 (0.28) | 0.001 |
| **Cardiac Parameters** |  |  |  |
| **AF [N, column %]** |  |  |  |
| No | 116 (73.89) | 230 (89.49) |  |
| Yes | 39 (24.84) | 24 (9.34) |  |
| Missing | 2 (1.27) | 3 (1.17) |  |
| Total | 157 (100) | 257 (100) | <0.0001 |
| **PFO [N, column %]** |  |  |  |
| No | 149 (94.9) | 246 (95.72) |  |
| Yes | 4 (2.55) | 6 (2.33) |  |
| Missing | 4 (2.55) | 5 (1.95) |  |
| Total | 157 (100) | 257 (100) | 0.9 |
| **Baseline LA echo dilated [N, column %]** |  |  |  |
| No | 63 (40.13) | 145 (56.42) |  |
| Yes | 73 (46.50) | 77 (29.96) |  |
| Missing | 21 (13.38) | 35 (13.62) |  |
| Total | 157 (100) | 257 (100) | 0.002 |
| **Baseline LA echo status [N, column %]** |  |  |  |
| Normal | 63 (40.13) | 145 (56.42) |  |
| Mildly dilated | 53 (33.76) | 65 (25.29) |  |
| Moderately dilated | 18 (11.46) | 9 (3.5) |  |
| Severely dilated | 2 (1.27) | 3 (1.17) |  |
| Missing | 21 (13.38) | 35 (13.62) |  |
| Total | 157 (100) | 257 (100) | 0.002 |
| **LA dilated at the end [N, column %]** |  |  |  |
| No | 60 (38.22) | 156 (60.70) |  |
| Yes | 77 (49.04) | 66 (25.68) |  |
| Missing | 20 (12.74) | 35 (13.62) |  |
| Total | 157 (100) | 257 (100) | <0.0001 |
| **LA status at the end [N, column %]** |  |  |  |
| Normal | 60 (38.22) | 156 (60.7) |  |
| Mildly dilated | 45 (28.66) | 47 (18.29) |  |
| Moderately dilated | 23 (14.65) | 12 (4.67) |  |
| Severely dilated | 9 (5.73) | 7 (2.72) |  |
| Missing | 20 (12.74) | 35 (13.62) |  |
| Total | 157 (100) | 257 (100) | <0.0001 |
| **Baseline valvulopathy [N, column %]** |  |  |  |
| Yes | 48 (30.57) | 50 (19.46) |  |
| No | 88 (56.05) | 170 (66.15) |  |
| Missing | 21 (13.38) | 37 (14.4) |  |
| Total | 157 (100) | 257 (100) | 0.034 |
| **Valvulopathy at the end [N, column %]** |  |  |  |
| Yes | 26 (16.56) | 29 (11.28) |  |
| No | 20 (12.74) | 54 (21.01) |  |
| Missing | 111 (70.7) | 174 (67.7) |  |
| Total | 157 (100) | 257 (100) | 0.052 |
| LVMI baseline [g/m², mean (SD)] | 52.3 (35.2) | 35.2 (20.8) | <0.0001 |
| **Neurological** |  |  |  |
| **Stroke at baseline [N, column %]** |  |  |  |
| No | 139 (88.54) | 232 (90.7) |  |
| Yes | 18 (11.46) | 24 (9.3) |  |
| Total | 157 (100) | 257 (100) | 0.34 |
| **Stroke (including TIA) at the end [N, column %]** |  |  |  |
| No | 118 (75.16) | 213 (82.88) |  |
| Yes | 39 (24.84) | 44 (17.12) |  |
| Total | 157 (100) | 257 (100) | 0.057 |
| **Stroke type at the end [N, column %]** |  |  |  |
| No stroke | 118 (76.43) | 213 (82.1) |  |
| TIA | 8 (4.46) | 6 (2.44) |  |
| Lacunar | 19 (11.46) | 26 (10.57) |  |
| Anterior | 5 (3.18) | 7 (2.85) |  |
| Posterior | 6 (3.82) | 5 (2.03) |  |
| Venous thrombosis | 1 (0.64) | 0 (0) |  |
| Total | 157 (100) | 257 (100) | 0.53 |
| **Stroke as a clinical event [N, column %]** |  |  |  |
| No | 135 (86) | 230 (89.5) |  |
| Yes | 22 (14) | 27 (10.5) |  |
| Total | 157 (100) | 257 (100) |  |
| Age of the first stroke [years, mean (SD)] | 49.8 (16.5) | 53 (17.9) | 0.4 |
| **Age of stroke and N215S status [years, mean (SD)]** |  |  |  |
| N215S | 66.4 (10.9) | 63.6 (15.9) | 0.0007 |
| No N215S | 45.39 (14.8) | 51.6 (17.8) | 0.0007 |
| **Baseline WML [N, column %]** |  |  |  |
| No WML | 87 (55.41) | 155 (60.31) |  |
| Fazekas 1 | 50 (31.85) | 70 (27.24) |  |
| Fazekas 2 | 15 (9.55) | 22 (8.56) |  |
| Missing | 5 (3.18) | 10 (3.89) |  |
| Total | 157 (100) | 257 (100) | 0.716 |
| **WML at the end [N, column %]** |  |  |  |
| No WML | 81 (51.59) | 142 (55.25) |  |
| Fazekas 1 | 127 (33.12) | 52 (29.18) |  |
| Fazekas 2 | 17 (10.83) | 30 (11.67) |  |
| Fazekas 3 | 4 (2.55) | 4 (1.56) |  |
| Missing | 3 (1.91) | 6 (2.33) |  |
| Total | 157 (100) | 257 (100) | 0.85 |
| **Acroparaesthesia [N, column %]** |  |  |  |
| Yes | 49 (31.21) | 56 (21.79) |  |
| No | 37 (23.57) | 88 (34.24) |  |
| Missing | 71 (45.22) | 113 (43.97) |  |
| Total | 157 (100) | 257 (100) | 0.028 |
| **Migraine [N, column %]** |  |  |  |
| No | 148 (94.27) | 231 (89.88) |  |
| Yes | 9 (5.73) | 26 (10.12) |  |
| Total | 157 (100) | 257 (100) | 0.12 |
| **Other** |  |  |  |
| **Angiokeratoma** |  |  |  |
| Yes | 47 (29.94) | 36 (14.01) |  |
| No | 39 (24.84) | 106 (41.25) |  |
| Missing | 71 (45.22) | 115 (44.75) |  |
| Total | 157 (100) | 257 (100) | <0.0001 |
| **Concomitant autoimmune disease [N, column %]** |  |  |  |
| No | 150 (95.54) | 243 (94.55) |  |
| Yes | 7 (4.46) | 14 (5.45) |  |
| Total | 157 (100) | 257 (100) | 0.8 |
| **Concomitant autoimmune disease, specific disease [N, column %]** |  |  |  |
| No | 150 (95.54) | 243 (94.92) |  |
| GCA vasculitis | 2 (0) | 0 (0) |  |
| Hypothyroidism | 2 (1.27) | 4 (1.56) |  |
| Leukocytoclastic | 0 (0) | 1 (0.39) |  |
| MS | 1 (0.64) | 0 (0) |  |
| Rheumatoid Arthritis | 1 (0.64) | 3 (1.17) |  |
| Sjogren Syndrome | 0 (0) | 2 (0.78) |  |
| Coeliac disease | 0 (0) | 1 (0.39) |  |
| Cryoglobulinemia type 3 | 0 (0) | 1 (0.39) |  |
| Polymyalgia Rheumatica | 1 (0.64) | 0 (0) |  |
| Scleroderma | 0 (0) | 1 (0.39) |  |
| Total | 157 (100) | 257 (100) | 0.4 |
| **Neoplasia [N, column %]** |  |  |  |
| No | 143 (91.79) | 237 (92.22) |  |
| Yes | 14 (8.92) | 20 (7.78) |  |
| Total | 157 (100) | 257 (100) | 0.6 |
| **Treatments** |  |  |  |
| **Initial Treatment [N, column %]** |  |  |  |
| No treatment | 12 (7.64) | 128 (49.81) |  |
| Agalsidase alpha | 97 (61.78) | 104 (40.47) |  |
| Agalsidase beta | 26 (16.56) | 18 (7) |  |
| Migalastat | 18 (11.46) | 6 (2.33) |  |
| Other | 4 (2.55) | 1 (0.39) |  |
| Total | 157 (100) | 257 (100) | <0.0001 |
| **Beta blocker treatment [N, column %]** |  |  |  |
| Yes | 17 (10.83) | 15 (5.84) |  |
| No | 66 (42.04) | 124 (48.25) |  |
| Missing | 74 (47.13) | 118 (45.91) |  |
| Total | 257 (100) | 157 (100) | 0.136 |
| **Statins treatment [N, column %]** |  |  |  |
| Yes | 31 (19.75) | 31 (12.06) |  |
| No | 52 (33.12) | 108 (42.02) |  |
| Missing | 74 (47.13) | 118 (45.91) |  |
| Total | 257 (100) | 157 (100) | 0.05 |
| **Antiplatelet treatment at the end [N, column %]** |  |  |  |
| No treatment | 104 (66.24) | 197 (76.65) |  |
| AAS | 38 (24.20) | 30 (11.67) |  |
| Clopidogrel | 9 (5.73) | 22 (8.56) |  |
| AAS + Clopidogrel | 2 (1.27) | 4 (1.56) |  |
| Missing | 4 (2.55) | 4 (1.56) |  |
| Total | 157 (100) | 257 (100) | 0.015 |
| **Anticoagulation at the end [N, column %]** |  |  |  |
| No treatment | 122 (77.71) | 235 (91.44) |  |
| Warfarine | 17 (10.83) | 13 (5.06) |  |
| Apixaban | 6 (3.82) | 1 (0.39) |  |
| Dabigatran | 3 (1.91) | 0 (0) |  |
| Rivaroxaban | 9 (5.73) | 8 (3.11) |  |
| Total | 157 (100) | 257 (100) | 0.001 |

Supplementary material 1: Characteristics of the full 414 cohort.
