# Supplementary material for: Stroke in Fabry Disease: Identification of Risk Factors for Stroke in a Large Single‐Centre Cohort
Source: Eur J Neurol. 2025 Nov 7;32(11):e70415. doi: 10.1111/ene.70415 (PMC12593542; doi:10.1111/ene.70415)
Supplement: Supplementary file 2 — Data S2: How to use and interpret the nomogram. [file ENE-32-e70415-s001.docx]

# **Supplementary material 2 – How to use the nomogram**

## **Acknowledgements**

The following guidance is adapted from the work of Dr A. Zlotnik and more information can be found in the following webpage:

<http://www.zlotnik.net/stata/nomograms/>

## **How to use the stroke-free survival nomogram**

1. Establish scores for all the variables in the nomogram from the clinical and laboratory data of the patient.
2. Obtain the total score.
3. At the bottom of the nomogram, obtain the survival (stroke-free probability) at a given age.
4. Use the age line closest to the patient real age.

## **Example**

“A 45 year old female affected with Fabry Disease has a mGFR of 55, no white matter lesions, no angiokeratoma, no acroparaesthesia, has a non-p.N215S genotype and has no concomitant autoimmune disease.”

The scores to add would be:

1. Angiokeratoma: 0
2. White matter lesions: 0
3. GFR more than 90: 4.75
4. p.N215S genotype: 10
5. Gender: 0
6. Autoimmunity: 0
7. Acroparaesthesia: 0

Total score: 14.75

The image below shoes how to get the scores.


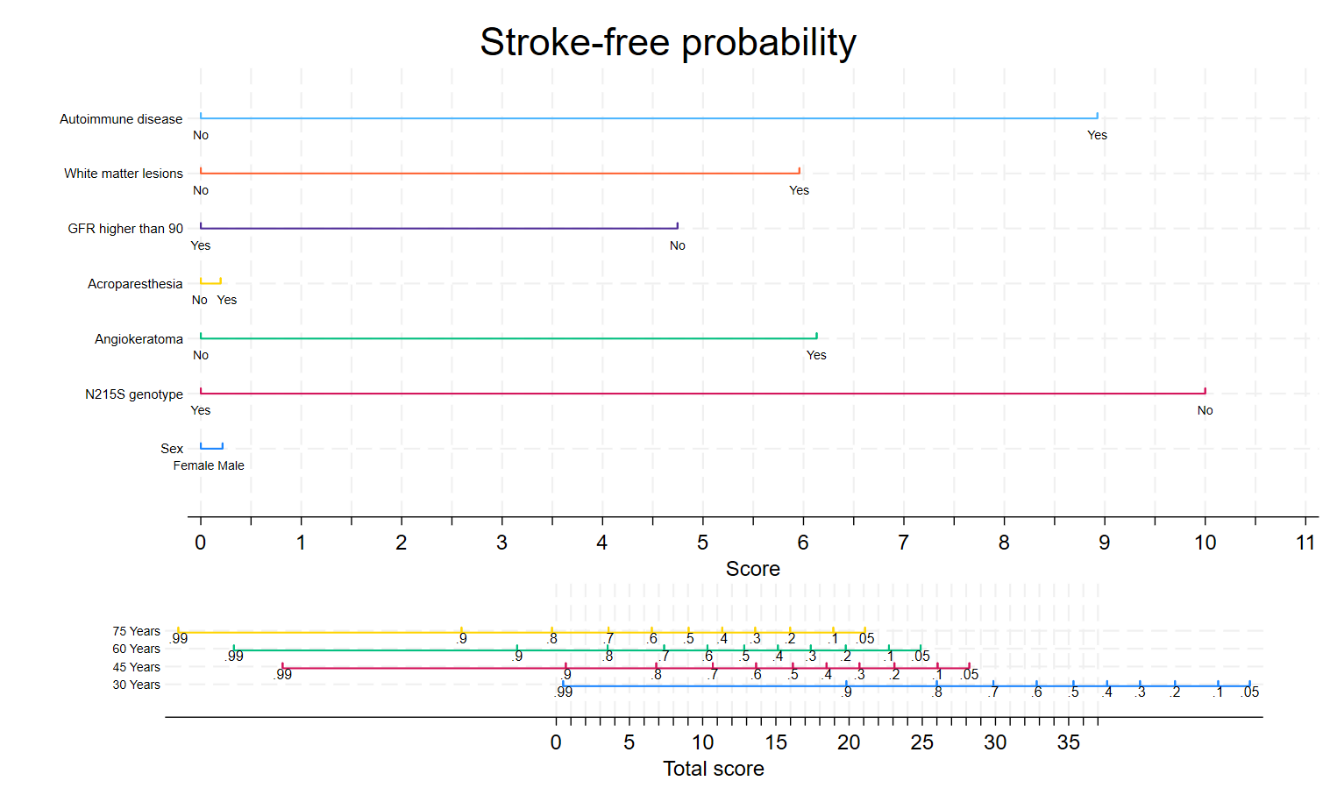


Then, the total score is used at the bottom scale to obtain the stroke-free survival at a given age.


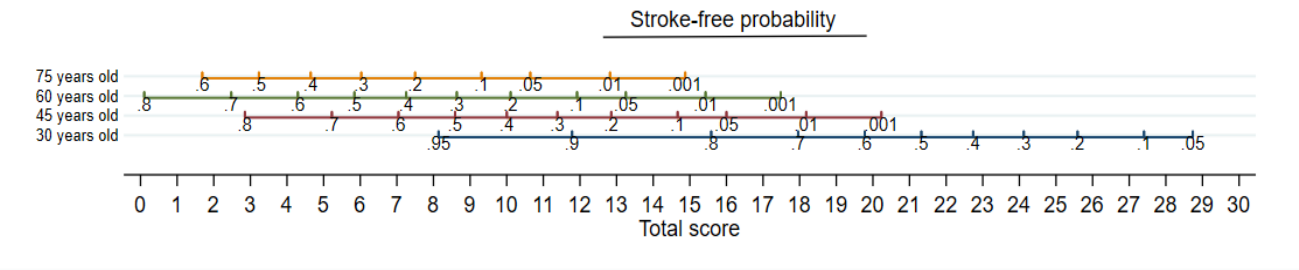


The stroke free survival with the given clinical characteristics at 45 years is approximately 11%. (Probability of having had a stroke 100-11: 89%)
