# Supplementary material for: Stroke in Fabry Disease: Identification of Risk Factors for Stroke in a Large Single‐Centre Cohort
Source: Eur J Neurol. 2025 Nov 7;32(11):e70415. doi: 10.1111/ene.70415 (PMC12593542; doi:10.1111/ene.70415)
Supplement: Supplementary file 3 — Data S3: Genetic variants of stroke patients. [file ENE-32-e70415-s004.docx]

| Genetic variant | Freq. | Percent | Cum. |
| --- | --- | --- | --- |
|  |  |  |  |
| A13P | 1 | 1.45 | 1.45 |
| A143T | 4 | 5.8 | 7.25 |
| C52G | 2 | 2.9 | 10.14 |
| E338K | 1 | 1.45 | 11.59 |
| Exon 1 del | 1 | 1.45 | 13.04 |
| G261V | 2 | 2.9 | 15.94 |
| G271S | 1 | 1.45 | 17.39 |
| G361R | 1 | 1.45 | 18.84 |
| I317T | 2 | 2.9 | 21.74 |
| I91T | 1 | 1.45 | 23.19 |
| L106F | 3 | 4.35 | 27.54 |
| L166P | 1 | 1.45 | 28.99 |
| L166S | 1 | 1.45 | 30.43 |
| M42V | 1 | 1.45 | 31.88 |
| N215S | 11 | 15.94 | 47.83 |
| N34D | 1 | 1.45 | 49.28 |
| P205T | 5 | 7.25 | 56.52 |
| Q107X | 1 | 1.45 | 57.97 |
| Q221P | 1 | 1.45 | 59.42 |
| Q321X | 1 | 1.45 | 60.87 |
| Q416P | 1 | 1.45 | 62.32 |
| R118C | 2 | 2.9 | 65.22 |
| R220X | 1 | 1.45 | 66.67 |
| R227X | 7 | 10.14 | 76.81 |
| R301P | 1 | 1.45 | 78.26 |
| R301Q | 1 | 1.45 | 79.71 |
| R301X | 3 | 4.35 | 84.06 |
| R342Q | 2 | 2.9 | 86.96 |
| R342X | 1 | 1.45 | 88.41 |
| R49L | 1 | 1.45 | 89.86 |
| c.1025delG | 1 | 1.45 | 91.3 |
| c.466delG (p.Ala156ProfsX9) | 1 | 1.45 | 92.75 |
| c.520delT | 1 | 1.45 | 94.2 |
| c.520delT | 1 | 1.45 | 95.65 |
| c.700_702 del GAT | 1 | 1.45 | 97.1 |
| c.700_702 het-del GAT | 1 | 1.45 | 98.55 |
| c.802-3_802-2 delCA | 1 | 1.45 | 100 |
|  |  |  |  |
| Total | 69 | 100 |  |

Supplementary material 3: Genetic variants of stroke patients
