# Supplementary material for: Stroke in Fabry Disease: Identification of Risk Factors for Stroke in a Large Single‐Centre Cohort
Source: Eur J Neurol. 2025 Nov 7;32(11):e70415. doi: 10.1111/ene.70415 (PMC12593542; doi:10.1111/ene.70415)
Supplement: Supplementary file 4 — Data S4: Stroke classification criteria. [file ENE-32-e70415-s003.docx]

**Supplementary data 4:**

In this study, stroke classification was performed according to the American Heart Association (AHA) guidelines published in 2013 ^1^. Briefly, the classifications are as follows:

1. Central Nervous System (CNS) infarction (brain, spinal cord, or retinal cell death attributable to ischemia based on imaging evidence of cerebral, spinal cord, or retinal focal ischemic injury in a defined vascular distribution)
2. Ischemic stroke (episode of neurological dysfunction caused by focal cerebral, spinal, or retinal infarction)
3. Silent CNS infarction (imaging evidence of CNS infarction, without a history of acute neurological dysfunction attributable to the lesion)
4. Intracerebral haemorrhage (focal collection of blood within the brain parenchyma or ventricular system that is not caused by trauma).

Strokes were classified according to AHA/ASA definitions. Within ischemic strokes, we further distinguished between lacunar (small-vessel pattern) and large-vessel distribution infarctions, the latter subcategorised by vascular territory (anterior vs posterior circulation). This hybrid classification reflects the recognised spectrum of Fabry-related neurovascular pathology, involving both small-vessel (manifested by lacunar infarcts, white matter hyperintensities, and microbleeds ^2 3^) and large-vessel abnormalities, particularly in the vertebrobasilar system, including dolichoectasia and increased posterior circulation arterial diameters ^4 5^. Although this is a controversial topic within the field, by combining infarct pattern and vascular territory, this scheme captures both major mechanisms of stroke in Fabry disease and aligns with prior large-scale observational studies, which have highlighted the diverse mechanisms of stroke in Fabry disease and their role in early-onset vascular disease ^6^.

Episodes compatible with a Transient Ischemic Attack (TIA), consisting of an episode of symptoms of brain ischemia which are clinically transient and without evidence of acute brain infarction, were also collected if reported in the medical records at any point by a neurology specialist. However, they were not included in the survival analysis to avoid bias. Stroke-mimics (including migraine and seizures) were collected when reported by the leading physician or a neurology specialist.

All MRI scans included in this study followed a standardised protocol including T1-weighted, T2-weighted, fluid-attenuated inversion recovery (FLAIR), diffusion-weighted imaging (DWI), apparent diffusion coefficient (ADC) mapping, and either susceptibility-weighted imaging (SWI) or gradient-echo (GRE) sequences.

According to the updated imaging-based definition of stroke by the AHA and the American Stroke Association (ASA), this study defines a stroke as any lesion consistent with ischemia observed on MRI sequences, including DWI, ADC, T2-weighted, and FLAIR images. This imaging strategy aligns with recommended protocols for stroke evaluation and enables the detection of cerebral infarction during acute, subacute, and chronic phases ^7^. DWI and ADC are highly sensitive for identifying acute ischemic changes, showcasing cytotoxic oedema within minutes of stroke onset, making them essential for the early identification of infarcts ^8 9^. T2-weighted and FLAIR sequences are useful in delineating subacute and chronic infarcts, characterising gliosis and white matter changes, and excluding alternative pathologies ^10 11^. SWI and GRE sequences are sensitive to paramagnetic blood products.

Furthermore, strokes were classified by vascular territory (e.g., anterior circulation, posterior circulation) based on clinical syndrome and MRI localisation. This method aligns with AHA/ASA guidelines ^1^, which emphasise identifying vascular territory as a critical step in determining stroke mechanisms.

Lacunar infarcts were defined as subcortical lesions ≤15 mm in diameter located in the territory of a deep perforating artery, without cortical involvement. They were identified using MRI sequences including DWI and ADC for acute infarcts, and FLAIR and T2-weighted imaging for subacute or chronic stages, consistent with AHA/ASA recommendations and established radiological criteria ^1 7^.

Moreover, white matter hyperintensities (WMHs) were assessed using FLAIR MRI and rated according to the Fazekas scale. This scale is well-validated and effectively reflects the burden of lesions in both periventricular and deep white matter ^12^. This approach is in accordance with the classification established by the AHA and ASA ^1^, which recognise WMHs as common ischemic changes associated with an increased risk of stroke, along with their pathological correlates seen on MRI. Haemorrhagic strokes were also analysed and differentiated as microbleeds versus intracerebral haemorrhage.

In conclusion, the stroke classification strategy used in this study aims to minimise detection bias by maximising sensitivity throughout all stages of infarction and reducing the likelihood of overlooking lesions due to variations in timing, lesion age, or imaging techniques.

**References:**

1. Sacco RL, Kasner SE, Broderick JP, et al. An updated definition of stroke for the 21st century: a statement for healthcare professionals from the American Heart Association/American Stroke Association. *Stroke* 2013;44(7):2064-89. doi: 10.1161/STR.0b013e318296aeca [published Online First: 20130507]

2. Cocozza S, Russo C, Pontillo G, et al. Neuroimaging in Fabry disease: current knowledge and future directions. *Insights Imaging* 2018;9(6):1077-88. doi: 10.1007/s13244-018-0664-8 [published Online First: 20181102]

3. Tapia D, Floriolli D, Han E, et al. Prevalence of cerebral small vessel disease in a Fabry disease cohort. *Mol Genet Metab Rep* 2021;29:100815. doi: 10.1016/j.ymgmr.2021.100815 [published Online First: 20211021]

4. Manara R, Carlier RY, Righetto S, et al. Basilar Artery Changes in Fabry Disease. *AJNR Am J Neuroradiol* 2017;38(3):531-36. doi: 10.3174/ajnr.A5069 [published Online First: 20170126]

5. Uceyler N, Homola GA, Guerrero Gonzalez H, et al. Increased arterial diameters in the posterior cerebral circulation in men with Fabry disease. *PLoS One* 2014;9(1):e87054. doi: 10.1371/journal.pone.0087054 [published Online First: 20140127]

6. Sims K, Politei J, Banikazemi M, et al. Stroke in Fabry disease frequently occurs before diagnosis and in the absence of other clinical events: natural history data from the Fabry Registry. *Stroke* 2009;40(3):788-94. doi: 10.1161/STROKEAHA.108.526293 [published Online First: 20090115]

7. Expert Panel on Neurological I, Ledbetter LN, Burns J, et al. ACR Appropriateness Criteria(R) Cerebrovascular Diseases-Aneurysm, Vascular Malformation, and Subarachnoid Hemorrhage. *J Am Coll Radiol* 2021;18(11S):S283-S304. doi: 10.1016/j.jacr.2021.08.012

8. Lovblad KO, Laubach HJ, Baird AE, et al. Clinical experience with diffusion-weighted MR in patients with acute stroke. *AJNR Am J Neuroradiol* 1998;19(6):1061-6.

9. Warach S, Gaa J, Siewert B, et al. Acute human stroke studied by whole brain echo planar diffusion-weighted magnetic resonance imaging. *Ann Neurol* 1995;37(2):231-41. doi: 10.1002/ana.410370214

10. Ay H, Buonanno FS, Rordorf G, et al. Normal diffusion-weighted MRI during stroke-like deficits. *Neurology* 1999;52(9):1784-92. doi: 10.1212/wnl.52.9.1784

11. Kidwell CS, Chalela JA, Saver JL, et al. Comparison of MRI and CT for detection of acute intracerebral hemorrhage. *JAMA* 2004;292(15):1823-30. doi: 10.1001/jama.292.15.1823

12. Fazekas F, Chawluk JB, Alavi A, et al. MR signal abnormalities at 1.5 T in Alzheimer's dementia and normal aging. *AJR Am J Roentgenol* 1987;149(2):351-6. doi: 10.2214/ajr.149.2.351
